# Treatment of an Advanced NSCLC Patient with a Rare OSBPL9-ALK Fusion: A Case Report

**DOI:** 10.3390/healthcare14183085

**Published:** 2026-09-19

**Authors:** Liang Chen, Hu Chen, Lei Feng, Zihao Zhang, Zhepeng Liu

**Affiliations:** 1 Department of Respiration, The First Affiliated Hospital of Wannan Medical University (Yijishan Hospital of Wannan Medical University), Wuhu 241000, China; channear@wnmc.edu.cn (L.C.); kunhaiguou@163.com (H.C.); 2 Department of Radiology, The First Affiliated Hospital of Wannan Medical University (Yijishan Hospital of Wannan Medical University), Wuhu 241000, China; fenglei20152015@163.com; 3 Department of Oncology, The First Affiliated Hospital of Wannan Medical University (Yijishan Hospital of Wannan Medical University), Wuhu 241000, China; 15722914559@163.com

**Keywords:** *ALK* fusion, adenocarcinoma, *OSBPL9-ALK* fusion, model prediction, anti-angiogenic drug

## Abstract

*ALK* rearrangements define an important molecular subtype of lung adenocarcinoma, and the rearranged *ALK* serves as a well-established oncogenic driver in this malignancy. The most common type of mutation is the *EML4-ALK* fusion. Meanwhile, rare fusions are also frequently discovered with regard to *ALK*. However, clinical efficacy varies substantially across rare *ALK* fusion variants due to their distinct biological features, and individualized drug selection guided by fusion architecture and tumor characteristics is therefore required. This case presents the entire treatment process of a female patient with advanced lung adenocarcinoma who had an *OSBPL9-ALK* fusion. The patient was diagnosed in early 2021; by referring to previous reports and model prediction with AlphaFold 2 and Autodock Pymol, the new second-generation ALK inhibitor Ensartinib was chosen. After 24 months, due to disease progression, she switched to the third-generation ALK inhibitor, Lorlatinib, after model prediction. After 15 months, for further disease progression, the anti-angiogenic drug, anlotinib, was added to the treatment plan. Unfortunately, 6 months later, the disease progressed further, and the patient chose supportive treatment for economic reason. Nine months later, the patient died. The presentation of this case will provide a reference guideline for the subsequent treatment of lung adenocarcinoma with the same fusion. Meanwhile, model-based prediction for ALK fusion targeted therapy can provide a predictive reference for drug selection in patients presenting similar *ALK* rearrangements in the future.

## 1. Introduction

Lung cancer, one of the most common malignant global diseases, has become a leading cause of cancer-related deaths. In 2024, 2.6 million new cases and 1.9 million deaths estimated worldwide were reported [1]. Based on pathologic characteristics, lung cancer can be categorized into small cell lung cancer (SCLC) and non-small cell lung cancer (NSCLC) [2,3,4]. Unlike SCLC, certain genomic alterations can always be detected in NSCLC, especially in adenocarcinoma—for example, EGFR (epidermal growth factor receptor), ALK (anaplastic lymphoma kinase) and ROS1 (c-ros oncogene 1) [5,6,7,8,9,10], which indicates the target therapy can be applied. According to previous studies, the mutation rate of *ALK* is appropriately 5% in lung adenocarcinoma, and non-smokers and younger patients are often burdened by the mutation. The *ALK* oncogene mainly involves the breakage and rearrangement of the *ALK* gene with other genes, with the most prevalent fusion in NSCLC involving the kinase domain of ALK fused with echinoderm microtubule-associated protein-like 4 (EML4). The *EML4-ALK* fusion protein can cause dimerization of the intracellular kinase domain of ALK, thereby activating the classic downstream oncogenic signaling pathway, which leads to tumor progression and worsens patient prognosis [11].

Besides the typical *EML4-ALK* fusion, many rare *ALK* fusion genes have been reported previously, such as *KIF5B-ALK*, *SMPD3-ALK*, and *PRKCE-ALK* [12,13,14]. In this work, we discovered a rare *OSBPL9-ALK* fusion gene in a patient with lung adenocarcinoma. Here, we report the whole treatment process; during the treatment, Ensartinib (2nd generation TKI inhibitor) and Lorlatinib (3rd generation TKI inhibitor), as well as anlotinib (anti-angiogenic drug). were strategically used individually or in combination.

## 2. Case Report

In April 2021, a 43-year-old Chinese female with no history of smoking but a history of pelvic fracture, due to pain in the lower back and both lower limbs lasting for more than 20 days, sought medical treatment at an outside hospital. She underwent a thoracolumbar magnetic resonance imaging (MRI) examination, and abnormal bone signals were scanned in T10, T11, and L4, along with compression fracture of the vertebral bodies, suggesting pathological fractures and potential metastasis. Due to the high possibility of metastatic lesions, further chest computed tomography (CT) was advised, the CT revealed a mass in the left lower lobe of the lung with mediastinal lymphadenopathy, and the preliminary diagnosis considered lung cancer with bone metastasis. Although the patient experienced a CT-guided percutaneous transthoracic needle biopsy (PTNB), the pathological result showed no evidence of malignancy.

**Figure 1:**
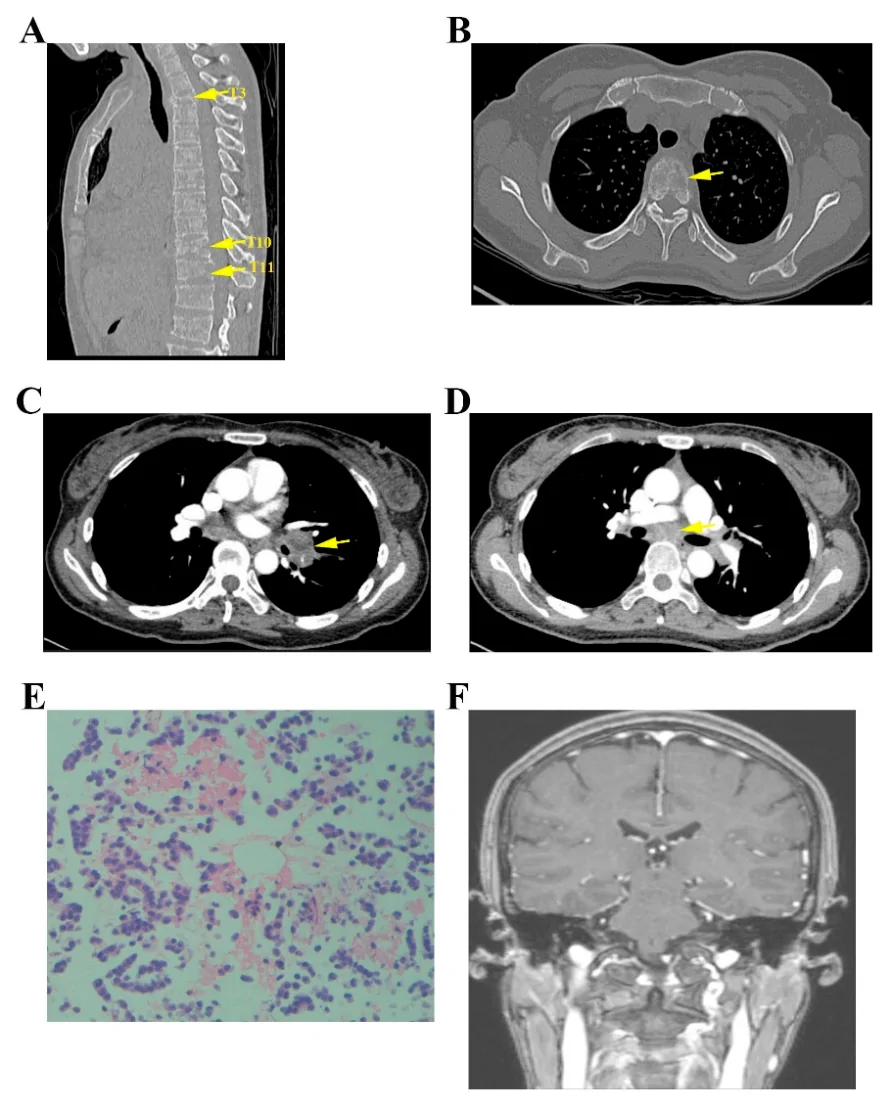
A female patient suffering from advanced lung adenocarcinoma identified by imaging and HE staining. The chest CT scan presents abnormal bone destruction along with compression fracture of the vertebral bodies (**A**,**B**). Meanwhile, a mass in the left hilum and mediastinal lymphadenopathy were detected (**C**,**D**). (**E**): HE staining of mediastinal nodes. (**F**): Brain contrast-enhanced MRI. T, thoracic vertebra. The lesion is indicated by yellow arrow.

In May 2021, she was admitted to our hospital with undiagnosed condition. After inquiring about her medical history, it was found that the patient had no cough or other respiratory symptoms; however, a slight breath sound decrease was detected in the left lung. Muscle strength in both lower limbs was assessed as grade 3 on the Medical Research Council scale, and further blood tests revealed elevated levels of alkaline phosphatase (ALP) at 318 µ/L (reference range, 35–135 µ/L) and carcinoembryonic antigen (CEA) at 12.62 ng/mL (reference range, 0–5 ng/mL), while no significant abnormalities were found in the complete blood count (CRC) and electrocardiogram (ECG). To confirm the pathology of the mass, further chest CT and endobronchial ultrasound-guided transbronchial needle aspiration (EBUS-TBNA) was performed; atypical epithelial cells were detected and further immunohistochemical staining (IHC) showed AE1/AE3(+), EMA(+), Napsin A(+), TTF-1(+), CK7(+), p40(-), LCA(-), Synaptophysin (partial+), CD56(-), CgA(-), and a Ki-67 (labeling index of approximately 10%), which indicated lung-derived adenocarcinoma with bone and lymph node metastasis (T3, T10, T11, mediastinal lymph node) (Figure 1A–E). However, no metastatic lesions were scanned in the brain via contrast-enhanced MRI (Figure 1F). The adrenal glands were not assessed. Therefore, the diagnosis was confirmed as left lower lobe adenocarcinoma, stage IVB (cT4N2M1c).

**Figure 2:**
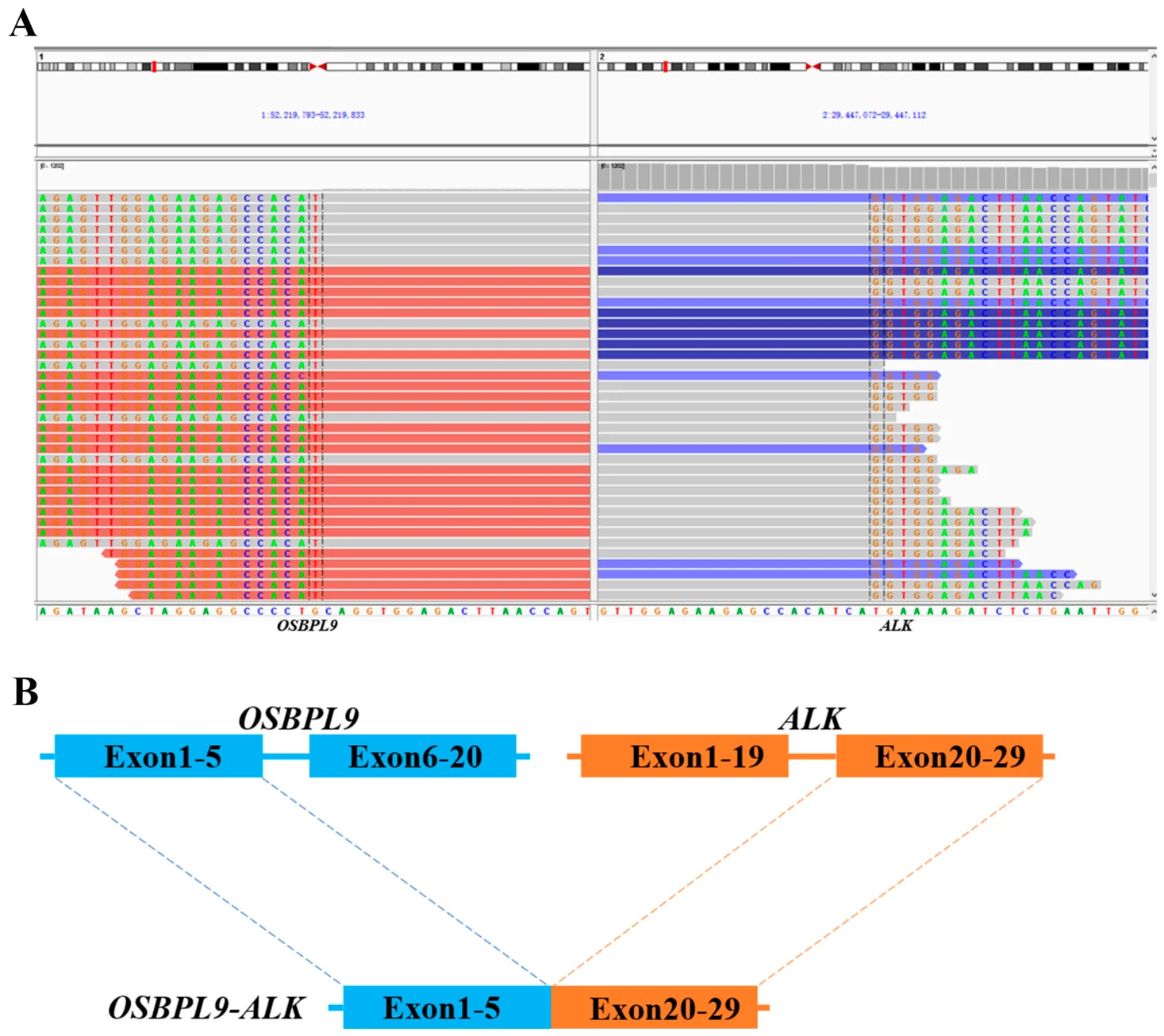
Schematic diagrams of *OSBPL9-ALK* fusion in the case. (**A**): DNA sequencing indicates *OSBPL9-ALK* mutation visualized by Integrative Genomic Viewer (IGV). (**B**): Schematic diagrams of *OSBPL9-ALK* mutation.

**Figure 3:**
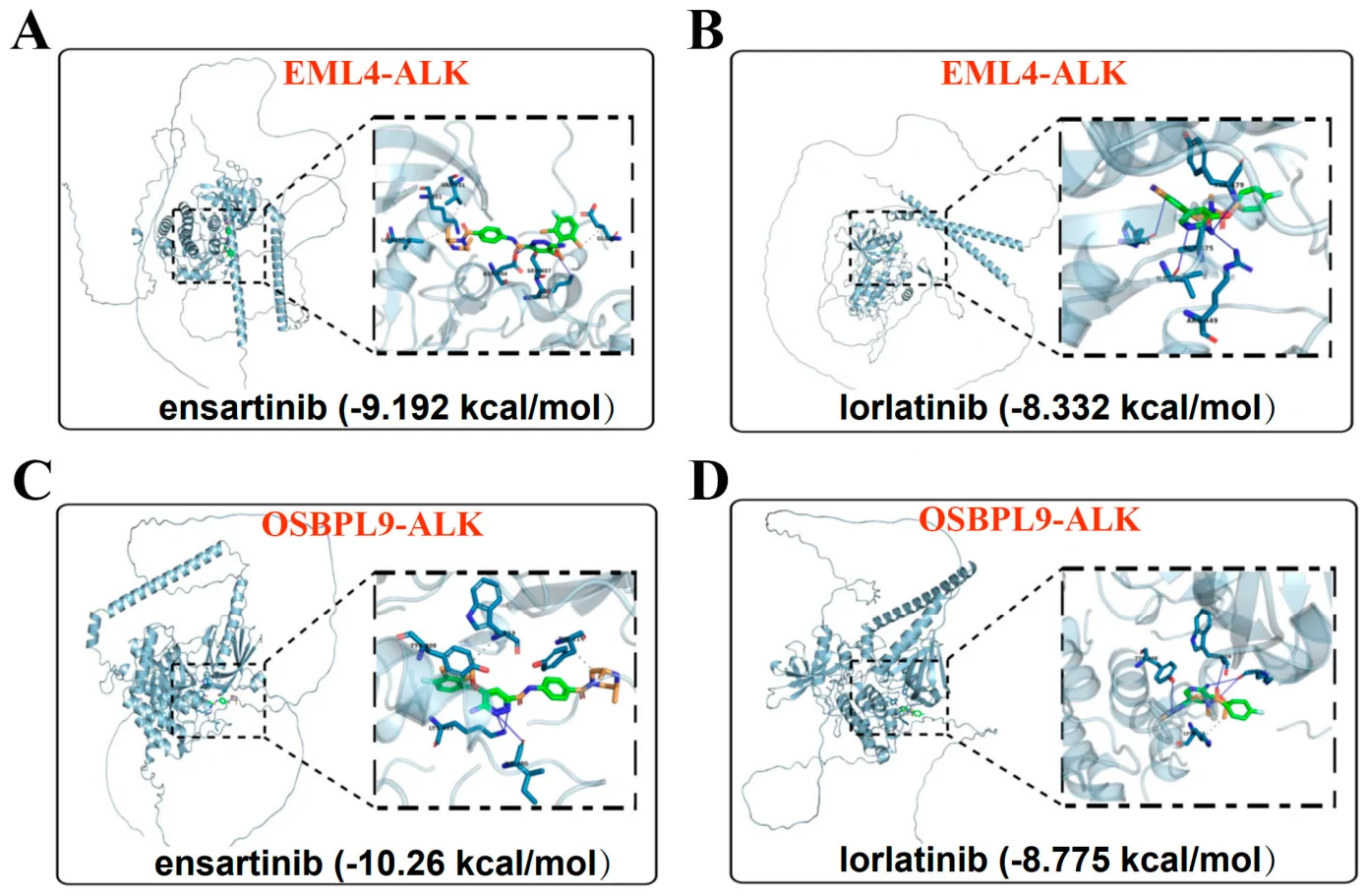
Molecular affinity prediction with regard to the most common *EML4-ALK* fusion or *OSBPL9-ALK* fusion with Ensartinib and Lorlatinib. To facilitate drug selection, with AlphaFold2 and Autodock Pymol, the affinity between the *EML4-ALK* (E6:A20) model and Ensartinib (**A**) or Lorlatinib (**B**) was predicted. The same prediction was also performed between *OSBPL9-ALK* (O5:A20) and Ensartinib (**C**) or Lorlatinib (**D**).

**Figure 4:**
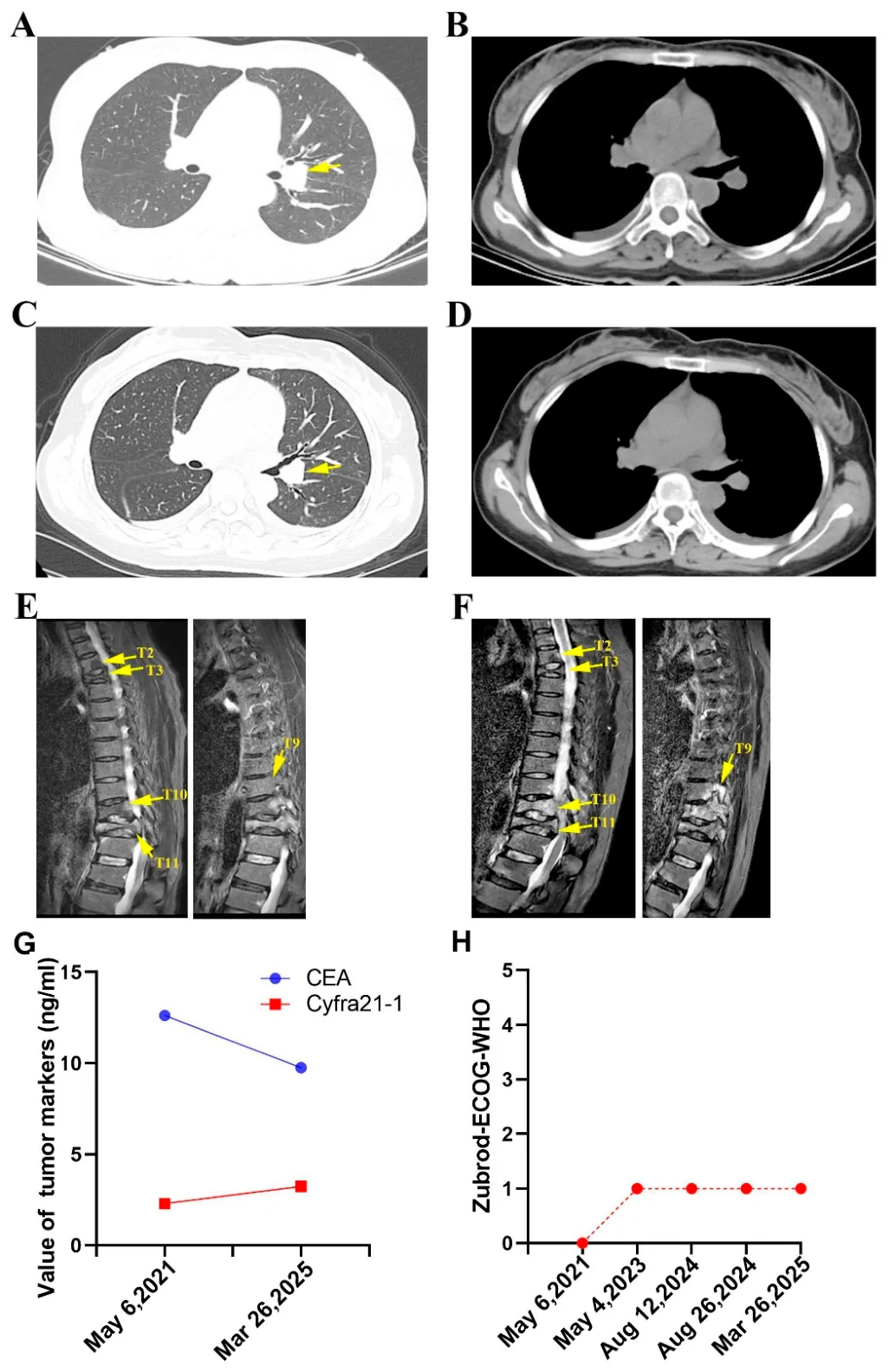
Treatment of the female patient with *OSBPL9-ALK* fusion harboring advanced adenocarcinoma. (**A**,**B**) The change in mass in the left hilum and mediastinal lymph node after treatment of Ensartinib for 3 months. (**C**–**E**) Disease progression after the treatment with Ensartinib for 24 months. (**F**): 15 months after Lorlatinib treatment. (**G**,**H**): Dynamic change in tumor markers and ECOG over multiple-line therapy. Reference normal value range of CEA, 0–5 ng/mL; normal range of Cyfra21-1, 0–1.8 ng/mL. The lesion is indicated by yellow arrow.

As recommended by clinical guidelines for adenocarcinoma, tissue specimens were sent for next-generation sequencing (NGS) in GENESEEQ (Nanjing, China) for molecular analysis with high-throughput sequencing platform (Supplementary File S1). While awaiting sequencing results, the patient was suffering from severe lower back pain and progressive weakness in the muscles of both lower limbs; we carried out an initial cycle of chemotherapy after the patient’s consent with AC (pemetrexed, carboplatin), accompanied by pain relief and other supportive treatments. The molecular analysis was reported in May 2021, and a rare *OSBPL9-ALK* fusion mutation was detected (Figure 2A,B). Owing to the local policy of charity drug donation and previous study [15], Ensartinib was focused. Meanwhile, to facilitate drug selection, with AlphaFold2 and Autodock Pymol, we predicated the molecular affinity between the most common *EML4-ALK* (E6:A20) fusion model and Ensartinib, as well as the *OSBPL9-ALK* fusion model and Ensartinib [16] (Figure 3A,C; Supplementary File S1). The results indicated that no matter whether in the *EML4-ALK* model or in the *OSBPL9-ALK* model, Ensartinib presents a likely affinity; furthermore, even a higher affinity was shown in model *OBSPL9-ALK*. Due to the results, Ensartinib, a second-generation ALK inhibitor, was selected for first-line therapy. The initial dosage of the drug was 225 mg/d, which was later reduced to 200 mg/d due to the occurrence of a rash. Three months after the treatment with Ensartinib, the pulmonary lesions and mediastinal lymph nodes decreased (Figure 4A,B), and the strength of the muscles in both lower limbs significantly increased. Concurrently, muscle strength in both lower limbs returned to normal. Hence, the treatment efficacy was assessed as a marked partial response (PR), and the inhibitor was continuously used.

In May 2023, 24 months after treatment with Ensartinib, the patient complained of recurrent lower back pain and muscle strength weakness in both lower limbs again. A chest CT scan was performed, but no visible change in pulmonary lesions or mediastinal lymph node lesions were scanned (Figure 4C,D). A further MRI scan showed increased metastatic bone lesions (T2, T3,T10,T11), revealing progression of disease (PD) (Figure 4E). Previous publications showed that Lorlatinib, a third-generation ALK inhibitor, could be an optimal option for next therapy measures [15]. Concurrently, the molecular affinity was also predicted, and the very same result was shown with regard to EML4-ALK/Lorlatinib and OSBPL9-ALK/Lorlatinib (Figure 3B,D), which implied that the Lorlatinib may also take effect on *OSBPL9-ALK*-fusion-suffering NSCLC patients. Furthermore, we chose Lorlatinib for the second-line therapy; after treatment with Lorlatinib, spinal pain was relieved and normal strength was restored in both lower limbs once more.

In August 2024, 15 months after Lorlatinib treatment, spinal metastases of the patient progressed again (T2, T3,T9,T10,T11), along with lower-limb muscle strength dropping to grade 1 (Figure 4F). Interestingly, certain tumor markers, as well as the physical condition score, presented no apparent discrepancy with previous examinations (Figure 4G,H), suggesting that stable serum tumor markers and patients’ unchanged ECOG-WHO performance status cannot exclude tumor progression; radiographic assessment remains essential. After comprehensive consideration, we recommended converting the therapy method to immunological therapy combined with chemotherapy, but the patient refused and chose to continue using Lorlatinib. Thus, in accordance with previous publications, oral anlotinib 12 mg once-daily was added following a 2-weeks-on/1-week-off cyclic schedule. No dose reduction or dose interruption related to anlotinib-associated adverse events (AEs) occurred during combination treatment [17].

In February 2025, the patient was hospitalized because of massive pleural effusion. Our multidisciplinary team (MDT) subsequently recommended that she discontinue Lorlatinib and anlotinib, and use immunotherapy or only supportive treatment; the patient chose the latter. Unfortunately, the patient passed away in October 2025 after treatment for 9 months.

## 3. Discussion

Our case observed the treatment process of a female patient with stage IV lung adenocarcinoma with *OSBPL9-ALK* fusion. The overall survival of the patient was 54 months. The patient was initially diagnosed in early 2021 and chose the new second-generation ALK inhibitor, Ensartinib. After 24 months, the disease progressed and the therapy regimen was switched to the use of Lorlatinib. After 15 months, unfortunately, the disease progressed further, and the anti-angiogenic drug, anlotinib, was added and combined with Lorlatinib. Six months later, the disease progressed comprehensively and the anti-tumor drugs were discontinued. After 9 months of supportive treatment, the patient passed away.

With regard to *ALK* fusion, *EML4-ALK* fusion accounts for more than 90% of *ALK* rearrangements, with v1 (E13;A20) and v3a/b (E6;A20) being the most common [18,19,20]. The patient harbors a *OSBPL9-ALK* fusion mutation, which is a rare *ALK* fusion subtype in lung adenocarcinoma. It belongs to the “diamond mutation” family and is formed by chromosomal translocation between the *OSBPL9* gene (oxysterol-binding protein-like 9 gene) and the *ALK* gene, resulting in a fusion gene that continuously activates downstream signaling pathways, driving tumorigenesis and development [15]. We selected Ensartinib for the first line due to charitable drug donation policies and recommendations from the relevant literature, as well as the model prediction. This patient experienced progression after using Ensartinib for 24 months, which was largely consistent with the results of previous related studies [21,22]. One case report described another rare *CEP44-ALK* mutation in lung atypical carcinoid, in which the first-line treatment was also Ensartinib, but the PFS was only 18.1 months; after progression, the patient was also subsequently treated with Lorlatinib and showed a favorable response [23]. Compared with this case, the response of *OSBPL9-ALK* fusion to Ensatinib was better than that of *CEP44-ALK* fusion. The OS of the patient we reported was 54 months, but no available reports of OS in randomized controlled clinical studies with regard to the first-line treatment of *ALK* mutation lung adenocarcinoma with Ensatinib can be found; however, a clinical study reporting an OS of 42.8 months for Ensatinib treatment after resistance to the first-generation drug crizotinib was found [24]. The prolonged OS of this patient compared to the previous one might be related to the subsequent use of the third-generation ALK inhibitor, Lorlatinib. After the onset of Ensartinib, the patient was switched to the third-generation ALK-TKI Lorlatinib, achieving a 15-month PFS, which is in line with the expected efficacy of Lorlatinib in second-line treatment [25]. Luo et al. recently reported a *KIF5B-ALK* (K15:A20) fusion, they experimentally used Lorlatinib for the first line. Interestingly, the patient acquired a partial response (PR) after 4 months, and the efficacy was kept stable at 8 months. Unfortunately, the PFS and OS were not reported further in the case [26]. After the second-generation ALK-TKI treatment progresses, approximately 56% of patients develop ALK kinase domain resistance mutations (such as G1202R, I1171N, L1196M, etc.). Lorlatinib, as the third-generation ALK-TKI, exhibits broad-spectrum and potent inhibitory activity against these common resistance mutations (especially G1202R). It is suitable for patients who may experience brain metastasis progression after Ensartinib treatment, so for patients who have progressed after the second-generation ALK-TKI, several guidelines recommend Lorlatinib as the preferred subsequent treatment option, which can effectively overcome most ALK-dependent resistance. As expected, 15 months later, the disease progressed again, which may stem from several reasons; after long-term treatment with Lorlatinib, the tumor may simultaneously receive ALK kinase domain mutations and bypass activation, resulting in a decline in the efficacy of single-agent treatment. At this time, combining anti-angiogenic drugs can exert a synergistic effect through different mechanisms. In addition, ALK-positive tumors have clonal heterogeneity. Some subclones may be insensitive to Lorlatinib and slowly proliferate to form new lesions. Combined treatment can cover more resistant subclones. Furthermore, the efficacy of Lorlatinib is related to the blood drug concentration. During long-term treatment, insufficient exposure may occur due to drug interactions or patient compliance issues, which will affect the efficacy. In our reported case, after the second disease progression, the anti-angiogenetic drug, anlotinib, was added, and the patient obtained an additional 6 months of disease control, demonstrating the role of anti-angiogenic drugs combined with targeted therapy in overcoming non-ALK-dependent resistance. Clinical studies have shown that ALK-TKI combined with anti-angiogenic drugs (such as bevacizumab) can significantly prolong PFS, especially in patients with slow disease progression. This combined strategy can be an effective means to delay drug switch. In this case, the patient experienced slow disease progression 15 months after monotherapy with Lorlatinib, which met the indications for the combination of targeted therapy with anti-angiogenesis, avoiding premature entry into the later line stage without standard treatment.

Unfortunately, due to cachexia and financial reasons, the patient did not receive immune checkpoint inhibitors (ICIs) and chemotherapy in the later stage. However, the patient still achieved a 54-month overall survival, which fully demonstrates the advantages of the treatment plan we chose earlier.

Interestingly, for the confirmation of characteristics of the lesion, the initial CT-guided percutaneous transthoracic needle biopsy yielded non-diagnostic findings, most likely caused by tumor sampling bias, given the peripheral location of the primary pulmonary lesion. The subsequent EBUS-TBNA of enlarged mediastinal lymph nodes successfully acquired tumor tissue for pathological and molecular confirmation.

It should be noted that the AlphaFold2 and Autodock Pymol were used during our clinical decision making for drug selection. However, the computational tools cannot replace wet-lab or clinical trail validation. In addition, all the docking scores have been presented with no replicates, no confidence of intervals and no comparison with the IC50 value from the available literature. Hence, it should not serve as definitive evidence for clinical drug choice.

## 4. Conclusions

In this case, we reported a patient suffering from advanced lung adenocarcinoma, who obtained a 54-month OS after an ALK inhibitor and subsequent anti-angiogenesis treatment. Our case has further confirmed that the *ALK* fusion gene is the “diamond mutation” of NSCLC. Through a rational sequential treatment with ALK-TKI, patients in the IV stage can also achieve a survival time close to that of a “chronic disease”. The therapy regimen from Ensartinib and Lorlatinib to sequential treatment with anti-angiogenic therapy maximized the benefits of targeted therapy and postponed the timing of chemotherapy usage. Also, our work further confirms the significance of precise detection; a biopsy combined with NGS testing can be conducted as a routine procedure, aiming at clarifying the mechanism of tumorigenesis or drug resistance, along with model prediction, guiding the selection of subsequent treatments, and avoiding the arbitrary change in medications. Taken together, we report a rare *ALK* rearrangement (*OSBPL9-ALK*) in a lung adenocarcinoma case and present all the processes of treatment from initial diagnosis to death with an impressive OS of about 54 months. Through our experience and mentioned model prediction, we provide a reference for the diagnosis and treatment of patients with the same fusion in the future.

## Data Availability

The datasets generated and analyzed during the current study are not publicly available due to patient-privacy related restrictions. Minimal de-identified datasets are available from the corresponding author upon reasonable request, subject to institutional ethical review.

## Supplementary Materials

The following supporting information can be downloaded at: https://www.mdpi.com/article/10.3390/healthcare14183085/s1, Supplementary File S1. Methods of molecular affinity prediction and NGS in the case.
