# Supplementary material for: Treatment of an Advanced NSCLC Patient with a Rare OSBPL9-ALK Fusion: A Case Report
Source: Healthcare (Basel). 2026 Sep 19;14(18):3085. doi: 10.3390/healthcare14183085 (PMC13606777; doi:10.3390/healthcare14183085)
Supplement: Supplementary file 1 [file healthcare-14-03085-s001.zip › healthcare-4356720-supplementary.pdf]

Method

Structural Modeling of ALK Fusion Proteins

Since the experimentally determined 3D structures of the full-length *EML4-ALK* and *OSBPL9-ALK* fusion proteins are not available in the Protein Data Bank (PDB), their structures were de novo modeled using AlphaFold 2. The full-length amino acid sequences of the wild-type EML4, OSBPL9, and ALK proteins were retrieved from the UniProtKB database. The chimeric sequences of the fusion proteins were digitally constructed based on their confirmed genomic breakpoints: *EML4-ALK* variant 3a (E6:A20) and *OSBPL9-ALK* (O5:A20). The constructed sequences were subsequently submitted to AlphaFold 2 for structural prediction. The quality of the generated 3D models was rigorously evaluated using the predicted Local Distance Difference Test (pLDDT) and the Predicted Aligned Error (PAE). Only the structural models demonstrating high confidence (pLDDT >80) within the ALK kinase domain, ensuring the geometric reliability of the ATP-binding pocket for subsequent drug binding, were selected for downstream molecular docking analyses.

Molecular Docking and Structural Visualization

The 3D structures of the *EML4-ALK* (E6:A20) and *OSBPL9-ALK* (O5:A20) fusion proteins were predicted using AlphaFold 2. The 3D chemical structures of the targeted inhibitors, Ensartinib and Lorlatinib, were obtained and subjected to energy minimization. Both the fusion proteins and the drug molecules were prepared using AutoDockTools to add polar hydrogen atoms, and compute Gasteiger charges, culminating in their conversion into the PDBQT format. Molecular docking was subsequently executed using AutoDock Vina to simulate the binding poses and calculate the thermodynamic binding affinities. To precisely enclose the ATP-binding pocket and allow adequate exploration of the local conformational space, the grid box dimensions were set to 25 × 25 × 25 Å. The coordinates for the *OSBPL9-ALK* fusion protein were set at X= -6.221, Y= 0.262, Z= -2.45, while for the *EML4-ALK* variant, the center was positioned at X= 5.662, Y= -6.212, Z= -4.236, ensuring a strictly controlled comparison for both Ensartinib and Lorlatinib within their respective kinase active sites. The docked conformation exhibiting the lowest binding energy was selected as the most thermodynamically stable model. Finally, the independent molecular graphics system PyMOL was employed to analyze the optimal spatial conformations and visualize the specific non-covalent interactions, including hydrogen bonding networks and hydrophobic contacts, between the inhibitors and the fusion kinase residues.

Gene Panel Sequencing

DNA extraction, library preparation, and targeted sequencing were performed in a CLIA- and CAP-accredited clinical laboratory. Genomic DNA was extracted from deparaffinized formalin-fixed, paraffin-embedded (FFPE) tissue, while leukocyte

genomic DNA from peripheral blood was used as the matched normal control. Following quality assessment of DNA concentration, purity, and fragment-size distribution, indexed sequencing libraries were constructed, enriched by hybrid capture using custom probes targeting 14 genes (*AKT1, ALK, BRAF, EGFR, ERBB2, KRAS, MAP2K1, MET, NF1, NRAS, PIK3CA, PTEN, RET, ROS1*), and sequenced on a high-throughput sequencing platform. Adapter-contaminated and low-quality reads were removed, and qualified reads were aligned to the human reference genome hg19 (GRCh37), followed by duplicate processing, local realignment, and base-quality recalibration. Single-nucleotide variants, insertions/deletions, copy-number alterations, and gene rearrangements were identified within the analytically validated reportable range and functionally annotated using databases including ClinVar, HGMD, LOVD, 1000 Genomes, ExAC, and gnomAD. Common population polymorphisms and variants of germline origin were excluded through population-frequency filtering and comparison with matched leukocyte DNA, and all candidate variants were manually reviewed using the Integrative Genomics Viewer (IGV).
